# Research to Strengthen Policy, Practice, and Advocacy on Housing for Aging Societies

**DOI:** 10.1093/geroni/igaa057.2492

**Published:** 2020-12-16

**Authors:** Emily Greenfield, Nancy Berlinger

## Abstract

Population aging alongside other global trends—such as urbanization, widening economic inequality, and climate change—accelerate the need for systematic efforts to improve housing for diverse individuals, families, and communities as they age. This symposium features gerontological research explicitly designed to advance policy, practice, and advocacy on aging and housing. The first presentation demonstrates the use of data from U.S. surveys to better characterize the nature of current and future challenges in access to affordable, accessible, and safe housing for older adults. The second paper presents findings from a mixed-methods action research project in Toronto involving tenants living in properties managed by the second largest senior housing provider in North America. The paper indicates how findings on facilitators and threats to aging in place directly inform policy implementation on integrated services in Toronto. The third paper presents findings from a longitudinal, in-depth interview study with leaders of age-friendly community initiatives in suburban New Jersey, demonstrating the simultaneous challenges and opportunities of embedding housing advocacy at the local level within broader age-friendly community change efforts. The fourth paper presents three case studies based on interviews with key stakeholders involved with anti-displacement housing preservation and public housing organizing in New York City, highlighting the often invisible work of older, lower income, African American women at the center of advocacy efforts to preserve affordable housing. Guided by interdisciplinary critical work on ethical responses to population aging, the discussant will integrate themes from the papers to propose a framework for research, policy, practice, and advocacy.

